# Beef-Tea

**Published:** 1847-12

**Authors:** 


*Beef-tea.*—When one pound of lean beef, free from fat, and separated from the bones, in the finely chopped state in which it is used for beef sausages or mince-meet, is uniformly mixed with its own weight of cold water, slowly heated to boiling, and the liquid, after boiling briskly for a minute or two, is strained through a towel from the coagulated albumen, and the fibrine, now becoming hard aad horny, we obtain an equal weight of the most aromatic soup, of such strength as cannot be obtained, even by boiling four hours, from a piece of flesh. When mixed with salt, and the other usual additions by which soup is usually seasoned, and tinged somewhat darker by means of rosted onions or burnt sugar, it forms the very best soup which can in any way be prepared from one pound of flesh.—*Liebig.*

